# Various Markers of Neuroendocrine Tumor: A Narrative Review

**DOI:** 10.7759/cureus.67493

**Published:** 2024-08-22

**Authors:** Sreetama Mukherjee, Sunita Vagha, Manish Mukherjee

**Affiliations:** 1 Pathology, Jawaharlal Nehru Medical College, Datta Meghe Institute of Higher Education and Research, Wardha, IND; 2 Pulmonary Medicine, R.G. Kar Medical College, Kolkata, IND

**Keywords:** illness, chromogranin a, prognosis, biomarkers, neuroendocrine tumors

## Abstract

Neuroendocrine tumors (NETs) are uncommon tumors that develop from specialized endocrine cells. Thyroid medullary carcinoma, phaeochromocytomas, pituitary tumors, carcinoid, and gastroenteropancreatic NET are just a few examples of the diverse group known as NET. In recent times, they have garnered significant interest due to their ease of palliation and ability to reveal the long-term impact of the specific hormone raised. Neuroendocrine indicators, particularly chromogranin A, are very helpful in the diagnostic process. Accurate biomarkers that can be employed for NET diagnosis, prognosis and follow-up, therapy stratification, and treatment response evaluation are greatly needed. Due to the great diversity of neuroendocrine neoplasms, particular biomarkers must be developed in order to diagnose, treat, and identify them. The several NET biomarkers covered in this review will aid in the fight against this uncommon illness.

## Introduction and background

Neuroendocrine (NE) neoplasms, or neuroendocrine tumors (NETs), are made up of cells within the NE system that can be found in several locations. Of these, the gastrointestinal tract and respiratory tract are the most often affected organ systems, accounting for 62-67% and 22-27% of all NETs, respectively. The digestive tract's small intestine (30.8%), rectum (26.3%), colon (17.6%), pancreas (12.1%), and appendix (5.7%) are the most often impacted areas by NETs. In the respiratory tract, lungs are the most common site of NETs accounting for 17-36% [1,2]. These tumor cells may release bioactive compounds into the bloodstream, typically in the form of peptide hormones or bioamines, which can result in a variety of clinical disorders, including the carcinoid syndrome. While the bulk of NETs are non-functional, this subset of NETs is referred to as functioning NETs. Despite the often slow progression of the condition, many people arrive with advanced stages of the illness [3]. Their distinctive morphologic patterns, immunohistochemical profile, and cytologic characteristics are what allow for their histological identification. Despite having a similar NE genesis, they differ greatly in terms of organ-specific traits, biological behavior, prognosis, and course of treatment. To make the diagnosis, discriminate between well-differentiated (WD) and poorly differentiated (PD) NETs, determine their rate of proliferation, forecast the prognosis, and choose the best course of treatment, pathological, immunohistochemical, genetic, and molecular markers are used [4,5].

Interest in the genetic susceptibility to several NETs has increased, especially because improved methods have made it easier to identify altered genes. Similar to this, through the examination of gene expression microarray profiles and multivariate analysis of complex features, important data can be used to prognosticate, and risk stratify patients. Numerous genes, including MEN1 and RET, have been linked to neuroendocrine tumorigenesis (NT); nonetheless, mutations in MEN1 continue to be the most prevalent genetic predisposition to NETs. Although there isn't any concrete proof, it's possible that NET is comparable to the well-researched colorectal carcinoma model, which involves a number of different genetic changes that activate oncogenes, inactivate tumor suppressor genes, or prevent apoptosis [6]. This review aims to address the challenge of diagnosis in patients with NETs, which significantly diminishes their quality of life and overall survival. By exploring biomarkers used for the early detection of NETs, this review seeks to highlight strategies for identifying patients at an earlier, more treatable stage of the disease.

## Review

Neuroendocrine tumors: associated pathology

The idea of NE differentiation in tumors is the release of bioactive substances, typically peptide hormones, into the bloodstream by the cancerous cells. Numerous NET and non-neoplastic NE cells share a histologic resemblance, which implies that tumors may develop from these mature counterparts. This method is likely very simplistic, despite considering well-developed neuroendocrine tumors (WD-NETs), which are the most similar to their normal cell counterparts [7]. The pathological definition of neuroendocrine differentiation is now recognized as the production of distinctive neurosecretory proteins that are immunohistochemically detectable, as well as architectural and cytological patterns resembling non-neoplastic neuroendocrine cells. The two most significant of them are synaptophysin and chromogranin A, while some experts also recognize that the expression of neuron-specific enolase (NSE) or neural cell adhesion molecule (CD56) is sufficient proof of neuroendocrine development [8]. Due to its lack of specificity, NSE's validity as a neuroendocrine marker has been called into question by several. Because of their common histologic characteristics, WD-NETs are therefore typically easily identified as having neuroendocrine differentiation in practice. This can be confirmed with widely accessible immunohistochemistry stains. In the past, the names "carcinoid tumor" (CT) or "islet cell tumor" were used to describe WD-NETs; however, in the gastroenteropancreatic system (GEPS) these labels have recently been replaced with "NET" to avoid the impression that a carcinoid tumor is a benign neoplasm. A CT or an islet cell tumor in the pancreas was the historical terminology for WD-NETs; however, in the GEPS, these terms have recently been replaced by NET to prevent the suggestion that a carcinoid tumor is a benign neoplasm [9]. Small and large cell NE carcinomas are the two main classifications for poorly-developed neuroendocrine tumors (PD-NETs), and they are separated by the size of the cell and a particular nuclear morphology.

The characteristics of small cell carcinoma on histology are sufficiently differentiable to enable diagnosis without the need for immunohistochemical demonstration of neuroendocrine differentiation; in contrast, large cell neuroendocrine carcinoma (LCNEC) requires immunological expression in at least one NE marker to differentiate it from other differentiated carcinomas. Among many other distinctions, the main one between PD-NETs and WD-NETs is their significantly higher proliferation rate [10]. Although they share several histologic characteristics linked to the neuroendocrine phenotype and neuroendocrine differentiation, mounting data indicates that WD-NETs and PD-NETs are, in fact, two distinct groups of neoplasms in the majority of organs. Particularly the WD-NETs, which have unique aetiologies in several organs, typically arise in individuals with NET syndromes, such as MEN1.

While PD-NETs are consistently extremely aggressive, WD-NETs vary in their level of aggression, with the majority being relatively indolent and having a natural history that can change over many years or decades. PD-NETs, particularly small cell carcinomas, demonstrate a notable but temporary susceptibility to platinum-based chemotherapy; in contrast, WD-NETs typically show no response to platinum or other cytotoxic treatments [11]. Furthermore, although these correlations are incredibly uncommon in WD-NETs, PD-NETs frequently develop in conjunction with exocrine-type precursor lesions or are mixed with components of squamous cell carcinoma (SCC) [12,13]. The difference between PD-NETs and WD-NETs is applicable in many organs, Despite the fact that classification schemes and terminology vary depending on the organ. For the majority of anatomic sites, grade-based predictive categorization has also been constructed, with the proliferative rate typically serving as the primary defining factor. The method used to calculate the proliferative rate is either counting the number of mitotic figures or, in some cases, the percentage of tumor cells that have immunolabeled for the proliferation marker Ki67 (also known as the Ki67 index) [14].

The World Health Organization (WHO) and the European Neuroendocrine Tumour Society (ENETS) have approved a single grading system that is currently widely used for NET, which comprises neoplasms of the entire gastrointestinal tract (GIT) and pancreas. This unification of grading parameters occurred recently. For many years, a separate WHO-accepted approach has been in existence for NET of the lungs and thymus in the thorax [15-17]. Unique characteristics unique to each place of origin are becoming apparent, and numerous other potential prognostic variables are being assessed. As a result, the final prognostic evaluation will probably include a grading system that changes with experience and is combined with additional prognostic information [18-20].

Neuroendocrine Markers “First Generation”

The immunohistochemical markers that are available for diagnostic use in pathology laboratories vary, but the most often used markers in the context of diagnosing a NET are SYP, NSE, and CgA. The sensitivity of SYP was found to be (86.5%), however, specificity was very less; the sensitivity and specificity for CgA were found to be (66.4%) and (70-100%) [21,22]. In many endocrine cells, CgA is necessary for the development of secretory granules and for the regulation of the exocytosis process. Its utility as an immunohistochemical marker for malignancies of the endocrine system was originally proven for parathyroid adenoma and many more, thus monoclonal antibodies were created specifically for this use [23,24]. In a similar vein, once SYP was discovered to be a vesicle protein (presynaptic) in a variety of nervous tissue, SYP expression was discovered in pancreatic islet cells, NETs, pheochromocytomas, and paragangliomas [25,26]. The expression of NSE and the cell adhesion molecule CD56 has also been discovered in a variety of NETs, however, the specificity is not ideal as an expression has also been observed in other tumors. In addition to these indicators of neuroendocrine differentiation, immunohistochemistry is useful in identifying the expression of distinct hormones associated with particular NE neoplasm forms, such as calcitonin in medullary thyroid cancer and serotonin in small intestine and appendiceal NETs [27,28].

Neuroendocrine Markers "Second Generation”

Since it was first discovered that NE and neurological cells selectively produced ISL LIM homeobox 1 (ISL1), research has shown that this transcription factor is essential for regulating the development of pancreatic endocrine cells [29]. ISL1 specifically binds the insulin gene promoter, which is essential for the NE cells of the Langerhans islets. Pathology-wise, insulinoma-associated protein (INSM1) has been shown to be a reliable NE neoplasm marker; pancreatic gynecological and pulmonary NETs, to mention a few, have positive immunoreactivity. Lastly, a calcium-binding protein called secretagogin (SECG) was formerly believed to be expressed only in Langerhans islets. Subsequent analyses, however, have shown that neuroendocrine carcinomas and NETs share very common expression patterns [30,31]. These markers may therefore express consistently in a biological setting even in the event that the NET downregulates its secretory machinery throughout the dedifferentiation process [32,33].

Biomarkers

The pathognomonic symptoms associated with functioning NETs can be caused by biomarkers and peptide hormones emitted by NETs. There are various biomarkers described so far such as catecholamines, calcitonin, glucagon, gastrin, pancreatic polypeptide, hydroxyindoleacetic acid (HIAA), etc. Urinary 5-hydroxyindoleacetic acid (5-HIAA) levels have been linked to several functional disorders, including carcinoid syndrome, insulinoma, glucagonomas, etc. We will discuss the biomarkers that have been most thoroughly examined for NET surveillance and diagnosis in this part. Interestingly, given that tissue sampling is now the most accurate way to diagnose a large number of malignancies. The value of biomarkers in the diagnosis of NETs is a topic of significant discussion [34].

CgA

The neurosecretory vesicles of NET contain the 49-kDa protein known as CgA. It can be detected by tissue immunostaining, serum, and blood plasma samples, and is a very specific marker for endocrine neoplasia patients [35-38]. Increased levels of CgA have been shown to be correlated with tumor load, the existence of metastases, and the effectiveness of treatment. One disadvantage of measuring CgA levels is the potential for elevated levels to arise from a variety of diseases or physiological modifications, including heart disease, pulmonary disease, food consumption, etc. The association between circulating CgA level and the tumor extent, overall survival, and tumor response prediction was assessed in a retrospective study involving 102 patients with gastroenteropancreatic NETs. After controlling for sex, stage, grade, primary site, and functionality, higher baseline CgA levels were linked to worse overall survival (OS) 95% confidence interval (CI), (1.06-172.47) [39].

SYP

Presynaptic vesicles contain SYP, which is a membrane glycoprotein that functions as an efficient biomarker for brain tissues. SYP has been connected to diabetic islet cell tumors, paragangliomas, and thyroid medullary carcinomas [40]. It is commonly found in tumors with varying degrees of differentiation and is believed to be a highly sensitive biomarker for the detection of neural epithelial cells; immunohistochemistry analysis can be used to investigate it [41]. The fact that this biomarker can be shown in a variety of malignancies, including non-small cell lung tumors and adrenocortical carcinoma, is a drawback [42,43]. Kriegsmann et al. retrospectively analyzed 1170 tissue samples and found that SYP was frequently expressed by lung adenocarcinomas and SCC [44].

5-HIAA

5-HIAA levels are typically higher in patients with metastatic midgut CT, especially in those with functional midgut NETs. 5-HIAA, which is present in urine, is the primary metabolite of serotonin [45,46]. A number of assays can be used to test urine 5-HIAA in less than 24 hours, but the most popular approach is high-performance liquid chromatography [47]. Patients who have persistent increases in their urinary 5-HIAA levels should have their 24-hour urinary serotonin evaluated, especially if their carcinoid illness is unclear. More than 300 patients who were referred to NET care were evaluated by Janson et al.; it was observed that urinary 5-HIAA levels were raised in 48% of those with bronchial CT and 76% of those with midgut NETs. Significantly increased urinary 5-HIAA levels were also seen in patients with midgut NETs and carcinoid heart disease [48-51].

Somatostatin Receptor

Cell surface somatostatin receptors (SSRs) are present in neuroendocrine cells. Once it attaches to the SSR, somatostatin affects cell division, neurotransmission, and hormone synthesis [52]. For SSR imaging, specific molecular targets are provided by SSRs. In-pentetreotide was the first effective application of SSR imaging, and it was crucial for identifying glomus paragangliomas and gastroenteropancreatic NETs [53-56].

Genes and prognostic biomarkers

Ki-67 Proliferation Index

The pathologic tissues of individuals who underwent pancreatic excision for pancreatic NET were stained by Hamilton et al. Researchers discovered that patients with a Ki-67 >9% had a worse OS rate and a significantly higher chance of illness recurrence [57-59]. Furthermore, a relation between Ki-67 levels and the reaction to treatment, including somatostatin analogs, has been found [60,61]. Additionally, it has been observed that Ki-67 is predictive of the outcomes of liver-directed therapy. In a multi-center retrospective study, Chen et al. discovered a strong correlation between a higher tumor grade and reductions in both overall and hepatic progression-free survival [62]. Furthermore, an analysis has been conducted comparing the Ki-67 scores and treatment outcomes among various embolic therapies. A retrospective study carried out at a single institution in patients of NETs found that yttrium-90 transarterial radioembolization was linked to a greater overall survival with a Ki-67 score ≥3% when compared to transarterial chemoembolization. This was not the case for patients with a Ki67 <3% [63,64].

CgA

Patients with NETs have a worse prognosis when their plasma CgA levels are high [65]. After evaluating 301 NET patients, Janson et al. found that plasma CgA of more than 5000 μg/l was a reliable indicator of a bad prognosis. Reductions in CgA levels following medication beginning have been linked to favorable treatment outcomes in a number of studies [66,67]. It has been observed that CgA decreases or returns to normal in tumors treated with liver-directed treatments, such as hepatic artery embolization (HAE) and radiofrequency ablation [68,69].

5-HIAA

It has been shown that 5-HIAA levels predict the progression of disease and whether patients with midgut NETs have liver metastases. Wedin et al. discovered in a prospective single-institution investigation that patients with WD-NETs had considerably higher serum 5-HIAA levels when they had liver metastases and/or extensive liver involvement [70]. Studies have revealed that the median survival of individuals with urine 5-HIAA >300 μmol/24 h is much lower than that of those with lower values. In addition, prognosis can be predicted by the rate of growth of urine 5-HIAA. The disease-specific death was considerably higher in those with small-intestine NETs of uncertain origins who had urine 5-HIAA doubling times <434 days compared to those with longer doubling times [71]. 5-HIAA levels have been reported to decrease following transarterial liver-directed therapy in a number of trials [72,73]. The effects of HAE in individuals with carcinoid syndrome were assessed by Carrasco et al. who found that all patients with objective imaging and clinical improvement had lower urinary 5-HIAA levels. Studies have shown that when HAE is combined with systemic medication, the effects on controlling carcinoid symptoms and reducing 5-HIAA levels are greater than when interferon therapy is administered alone [74].

## Conclusions

The evaluation of established biomarkers such as CgA, 5-HIAA, and Ki-67, along with newer tests like the NETest and genomic sequencing, has significantly improved the prognostication and treatment of NETs. These advances facilitate more personalized treatment options, leading to potentially better patient outcomes. Ongoing research into these areas holds promise for the development of more accurate diagnostic tools and therapeutic strategies, ultimately aiming to improve disease diagnosis, prognosis, and treatment for NET patients.
